# Reduced bioenergetics and toll-like receptor 1 function in human polymorphonuclear leukocytes in aging

**DOI:** 10.18632/aging.100642

**Published:** 2014-03-03

**Authors:** Feng Qian, Xiuyang Guo, Xiaomei Wang, Xiaoling Yuan, Shu Chen, Stephen E. Malawista, Linda K. Bockenstedt, Heather G. Allore, Ruth R. Montgomery

**Affiliations:** ^1^ Department of Internal Medicine, Yale University School of Medicine, New Haven, CT 06520, USA; ^2^ State Key Laboratory of Genetic Engineering and Ministry of Education Key Laboratory of Contemporary Anthropology, School of Life Sciences, Fudan University, Shanghai, 200433, China; ^3^ Roger Williams Medical Center, Boston University School of Medicine, Providence, RI 02908, USA

**Keywords:** neutrophils, Toll-like receptors, p38 Map kinase signaling, aging, bioenergetics, integrins

## Abstract

Aging is associated with a progressive decline in immune function (immunosenescence) resulting in an increased susceptibility to viral and bacterial infections. Here we show reduced expression of Toll-like receptor 1 (TLR1) in polymorphonuclear leukocytes (PMN) and an underlying age-dependent deficiency in PMN bioenergetics. In older (>65 years) adults, stimulation through TLR1 led to lower activation of integrins (CD11b and CD18), lower production of the chemokine IL-8, and lower levels of the phosphorylated signaling intermediate p38 MAP kinase than in PMN from younger donors (21-30 years). In addition, loss of CD62L, a marker of PMN activation, was reduced in PMN of older adults stimulated through multiple pathways. Rescue of PMN from apoptosis by stimulation with TLR1 was reduced in PMN from older adults. In seeking an explanation for effects of aging across multiple pathways, we examined PMN energy utilization and found that glucose uptake after stimulation through TLR1 was dramatically lower in PMN of older adults. Our results demonstrate a reduction in TLR1 expression and TLR1-mediated responses in PMN with aging, and reduced efficiency of bioenergetics in PMN. These changes likely contribute to reduced PMN efficiency in aging through multiple aspects of PMN function and suggest potential therapeutic opportunities.

## INTRODUCTION

Aging is associated with a progressive decline in immune function -- termed immunosenescence -- that results in an increased susceptibility to viral and bacterial infections and decreased response to vaccines [1]. The adaptive immune system is heavily affected in aging, with a well-documented decline in humoral as well as cell-mediated immune responses [2]. Aging of the innate immune system is complex in spanning multiple cell types, activation states, and tissue context, and, while incompletely understood, it is characterized by dysregulation and inappropriate persistence of inflammatory responses [1]. Studies of the cellular mechanisms of aging broadly suggest roles for the accumulation of reactive oxygen species (ROS) leading to damage of biomolecules [3], an age-associated decrease of autophagy that reduces clearance of damaged mitochondria and cellular proteins [4], an NF-κB-dependent inflammatory state in the hypothalamus leading to immune-neuroendocrine decline [5], and dysregulation of glucose metabolism that may underlie many aspects of senescence and aging-related diseases [6]. Indeed, studies of caloric restriction in many species have been shown to extend lifespan [7].

Short-lived polymorphonuclear leukocytes (PMN), the most numerous cells in the human innate immune system, are newly released each day from bone marrow precursors and yet show effects of aging [8]. PMN are the first immune cells to migrate to pathogen-infected sites, and release potent reactive oxygen and nitrogen intermediates, along with granules containing abundant antimicrobial peptides, and more recently have been shown to play a role in cytokine production, extracellular trap formation, and regulation of adaptive immunity [8]. Deficiencies of PMN functions in aging include reduced recruitment, phagocytosis, release of cytokines and granules, and diminished microbial activity. These comprehensive deficiencies suggest an age-related dysfunction in signal transduction [9-12].

Antimicrobial host defense responses are triggered by Toll-like receptors (TLRs), pattern recognition receptors that recognize conserved molecular patterns on microbes [13, 14]. We have recently shown the effects of aging on TLR expression and function in innate immune cell types of a large cohort of younger and community-dwelling older adults. Age-associated deficits in monocytes include reduced surface expression of TLR1, reduced TLR1/2 function, reduced costimulatory responses associated with reduced vaccination efficiency [15, 16], and elevation in TLR5 that may contribute to the heightened ‘inflammaging” milieu [17]. Decreases in TLR expression and function in primary human dendritic cells were strongly associated with poor antibody response to influenza immunization [18] and reduced production of type I IFN to infection with West Nile virus [19], and macrophages from older adults showed dysregulation of TLR3 in response to infection with West Nile virus [20]. We have undertaken the present study to examine the age-related alterations of expression and function of TLRs on PMN, and investigate underlying mechanisms that may contribute to the impaired immunity observed in older adults.

## RESULTS

### Effect of aging on expression of TLRs in human PMN

To identify effects of aging on PMN, we recruited younger (N=38) and older (N=40) healthy individuals to compareTLR expression and function. Subjects were 57% female and 84% white (Table 1). PMN are short-lived cells that rapidly undergo apoptosis in vivo (8-12 hours), and in vitro may release highly active hydrolytic enzymes from their granules, which may degrade surface proteins [21]. In consideration of the special features of PMN, several measures were undertaken to maximize the reproducibility of our findings. Only samples that could be assessed within 2 hr of the blood collection were used, and to reduce the time to measurement, the expression of TLRs on PMN was assessed by flow cytometry using a whole blood assay. We found no significant difference in the percentage of PMN with age (data not shown), in keeping with previous reports [21].

**Table 1:** Participant characteristics

	Young (N=38)	Old (N=40)	Total (N=78)	P-value*
Age (y), mean(SD) (range)	25.9(2.5) 22-30	74.5(7.0) 65-90	50.8(25.0) 22-90	-
Female gender, N (%)	21(55.3)	24(60.0)	45(57.7)	0.82
Race, N (%)				
White	29(76.3)	37(92.5)	66(84.6)	0.02
Black	1(2.6)	2(5.0)	3(3.9)	
Other	8(21.1)	1(2.5)	9(11.5)	
Hispanic	2(5.3)	1(2.5)	3(3.9)	0.61

* The p-values were calculated based on Fisher exact tests for categorical

**Figure 1:**
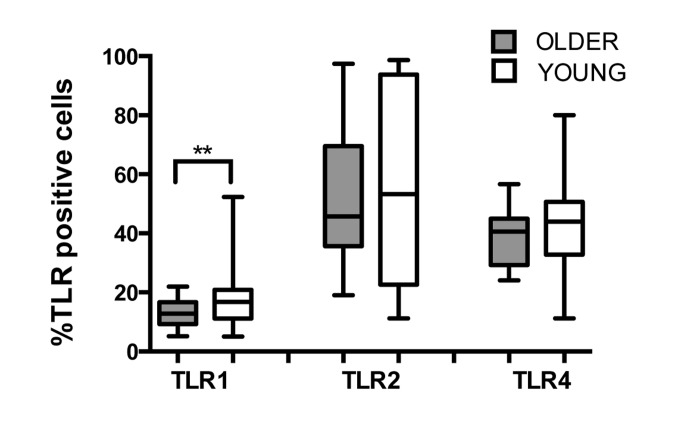
Effect of aging on expression of TLRs in human PMN Whole blood of younger (n=31) and older (n =22) adults was labeled for flow cytometry with lineage markers and TLRs at 4°C for 30 min following RBC lysis. Labeling was detected by LSR II. Data shown are % positive neutrophils for TLR surface expression. Values indicate the means ± SEM in young and older adults. Asterisks indicate statistical significance between younger and older cohort (Unadjusted t-test accounting for unequal variances, p < 0.02).

Human PMN express most TLRs (except TLR3) and respond to TLR ligand stimulation with production of pro-inflammatory cytokines and release of O_2_-, reflecting their active functions in the innate immune response [22]. We compared TLR expression between young and older adults and observed reduced expression of TLR1 by PMN of the older as compared to young adults (Fig. 1; p=0.02), which is consistent with previously described lower expression of TLR1 on monocytes and dendritic cells of older adults [15, 18]. Expression of TLR2 and TLR4 were equivalent in both age groups, as reported previously [23]. While TLR1 surface expression was reduced in PMN from older donors, no significant age-related differences were detected for mean fluorescent intensity of TLRs (data not shown), suggesting that the reduction in TLR1 surface expression may reflect age-related effects on intracellular trafficking pathways of TLR1, including changes in plasma membrane viscosity [24] or in PMN lipid raft domains in older individuals [25].

### TLR1 stimulated activation of PMN and cytokine production is reduced in older adults

**Figure 2:**
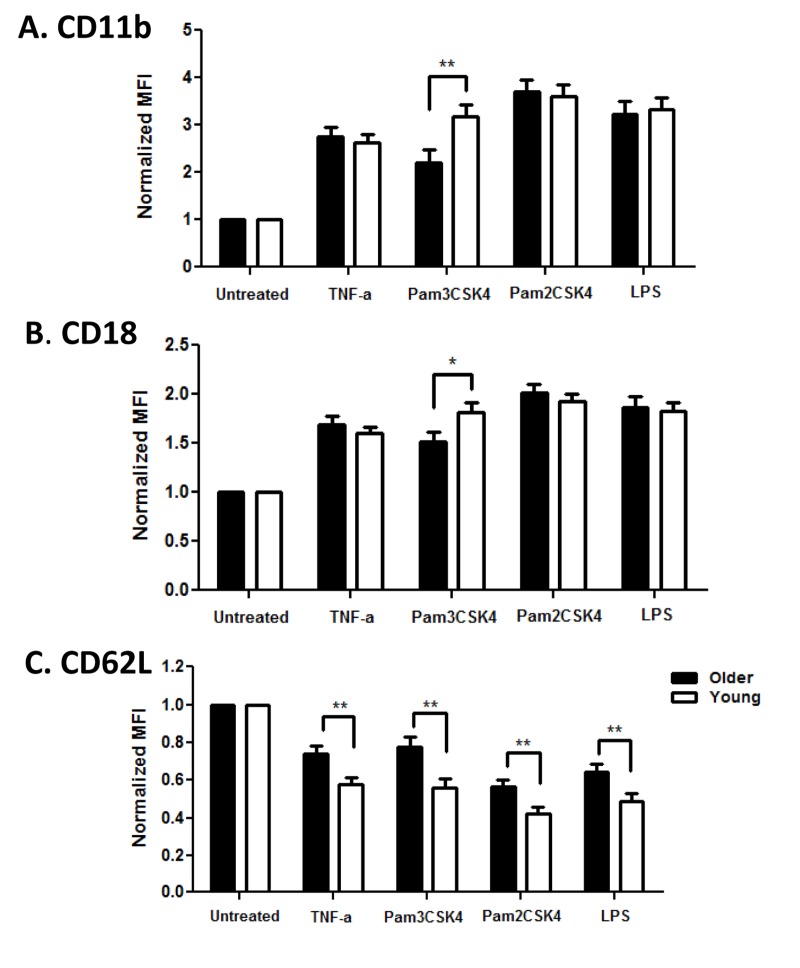
Age-associated alterations in PMN surface markers Whole blood of younger (n=32) and older (n =35) adults was stimulated with indicated ligands for 0, 15 min at 37 °C. Following RBC lysis, PMN were labeled for PMN markers at 4°C for 30 min. Flow cytometric labeling was detected by LSR II. Data shown are normalized MFI (Mean Fluorescence Intensity) of CD11b, CD18 and CD62L surface expression on PMN. Values indicate the means ± SEM in younger and older adults; asterisks indicate statistical significance between younger and older cohort (*P<0.05, **P < 0.01). For normalized CD11b and CD18 all stimulated PMN increased over time (P<0.001) and for CD62L all stimulated PMN decreased over time (P<0.001).

Activation of PMN leads to dramatic changes in adhesion to endothelial surfaces to promote diapedesis and PMN exit to the site of injury or infection, mediated in part by upregulation of integrins CD11b and CD18 and shedding of L-selectin (CD62L) from the cell surface [26]. We quantified expression of these adhesion markers to detect age-related differences in PMN functional efficiency. Baseline levels of CD11b and CD18 were not different between age groups, while CD62L was somewhat lower in the older cohort (data not shown). After stimulation with TLR ligands, PMN of both age groups upregulated CD11b and CD18, but the increase was significantly lower in PMN from older adults after stimulation through TLR1 (Pam3CSK4; Fig. 2A CD11b, p<0.01; Fig. 2B CD18, p<0.05). Notably, loss of CD62L expression after activation was also reduced on PMN from older adults, but this reduction was not limited to TLR1 stimulation (Fig 2C, p<0.01).

**Figure 3:**
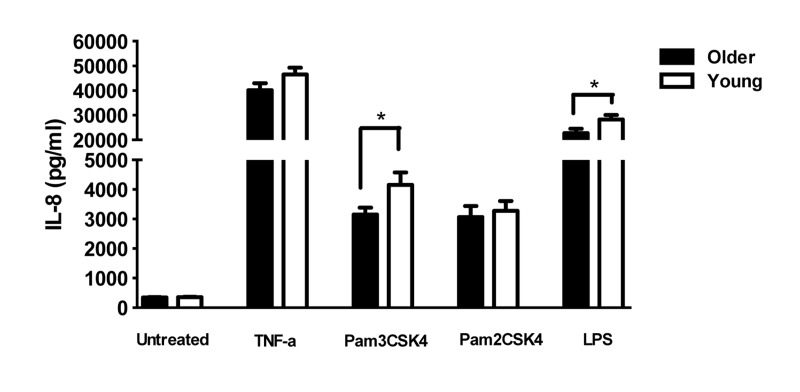
Effect of aging on TLR-stimulated production of cytokines by PMN In unstimulated vs TLR ligand-stimulated cells, the difference in the production of IL-8 in supernatants of PMN from younger (N=36) and older (N=34) adults. IL-8 was detected by ELISA after stimulation as shown. Values indicate the mean ± SEM concentration of IL-8 produced by PMN. Asterisks indicate statistical significance between younger and older cohorts (p < 0.05).

To determine the consequences of the lower levels of TLR1 on PMN in older adults, we quantified the production of cytokines from PMN of younger and older adults after stimulation by TLR ligands. PMN stimulated with ligands for TLR1 (Pam3CSK4) produced significantly higher IL-8 compared to PMN in medium alone, as expected [22],but levels from elderly adults were significantly lower than those from younger adults (Fig 3; p <0.05). Lower TLR1-induced cytokine production would be expected as a consequence of age-related reduced TLR1 expression. In contrast, stimulation with Pam2CSK4, the ligand for TLR2, elicited equivalent levels of IL-8 from PMN of both age groups (Fig. 3), likely reflecting the equivalent levels of TLR2 across age groups. Of note, production of IL-8 by PMN from older adults was also reduced after stimulation with LPS, the ligand for TLR4 (Fig. 3).

### Mechanism of reduced signaling by PMN

**Figure 4:**
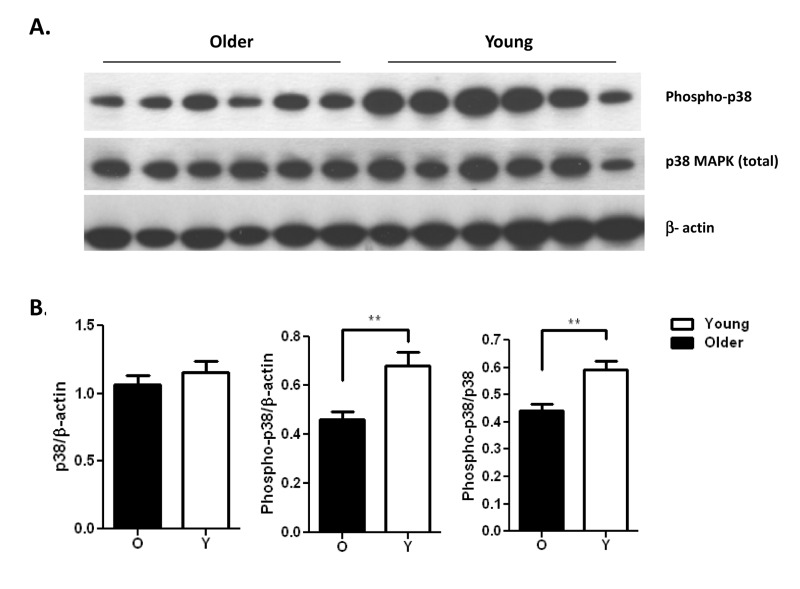
Pam3CSK4 stimulated neutrophils from the young show increased phosphorylation of p38 MAPK (**A**) immunoblot of total p38 MAPK and active phosphorylated p38 in a representative result of neutrophils from younger and older adults after stimulation with the TLR1/2 ligand Pam3CSK4 for 15 min. (**B**) Densitometry of immunoblot of p38 and phospho-p38 in neutrophils from 21 pairs of younger and older subjects after stimulation with Pam3CSK4 for 15min. Densitometry shows the means ± SEM of the ratio of total p38 and phospho-p38 to β-actin, and of phospho-p38 to total p38. Asterisks indicate statistical significance between younger and older cohort (** p < 0.01).

Reduced activation and production of cytokines by PMN in elderly adults may be significant for multiple PMN functions in vivo that depend on adhesion for recruitment and cytokines for activation of interacting immune cells [8]. To dissect the mechanism(s) underlying this reduction, we examined key components of the signaling cascade of PMN activation to assess age-related changes in signaling pathways. Signaling intermediates in activation of PMN include MAP kinase and NF-κB pathways [27, 28] and we have previously shown an age-dependent reduction in p38 MAPK pathway in TLR5 mediated production of IL-8 by monocytes [17]. Thus we assessed activation of p38 MAPK, signaling intermediate in PMN, and quantified PMN of both age groups for the level of phosphorylation of p38 MAP kinase after 15 min stimulation with the TLR1 ligand Pam3CSK4. While the total levels of p38 MAP kinase were not significantly different between age groups (Fig. 4A, middle row; ns), after stimulation the level of active phosphorylated p38 was significantly elevated in PMN of younger adults (Fig. 4A, top row). Quantitation of densitometry of blots from 21 pairs of younger and older subjects comparing the relative level of phosphorylated to total p38 shows significantly more activation for PMN of younger adults (Fig. 4B; p<0.01).

### Reduced TLR1 mediated extension of PMN lifespan and cellular bioenergetics in older adults

**Figure 5:**
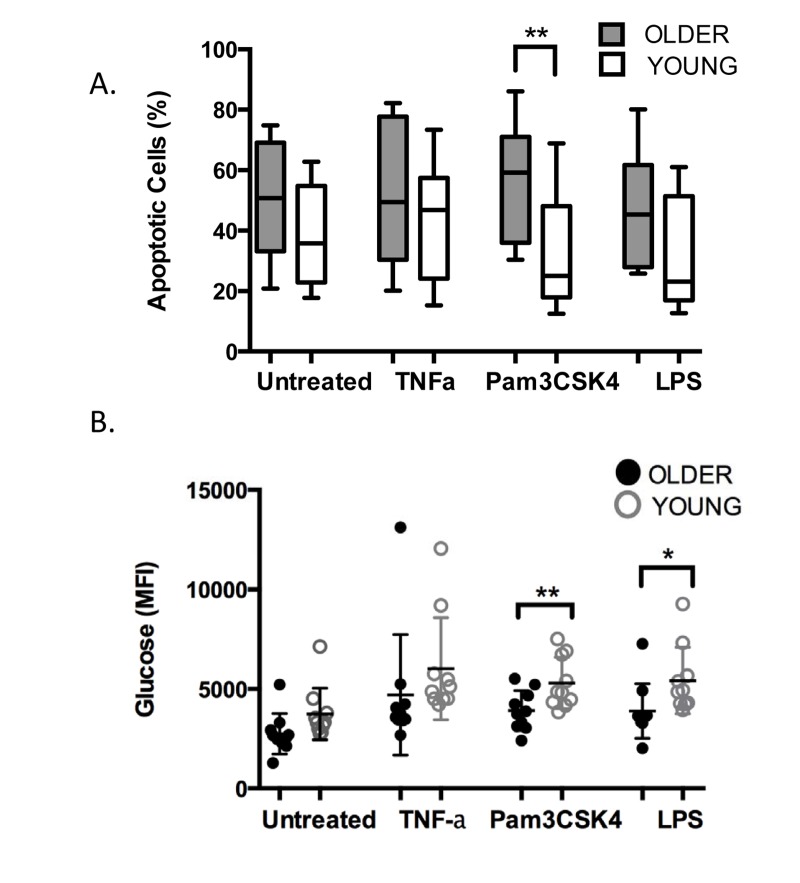
Age-related alterations in TLR1-mediated stimulation of PMN PMN from younger and older adults (10^6^/ml, n=10/group) were stimulated with TNF-α (20ng/ml), LPS (0.5μg/ml), or Pam3CSK4 (5μg/ml) in RPMI/10% human serum. Cells were incubated for 18 hr and labeled with Annexin V and PI for detection of apoptosis by flow cytometry (**A**). The level of fluorescent glucose (MFI of 2-NBDG) was quantified from aliquots of the same PMN by flow cytometry after 2 hr (**B**). Values indicate the mean ± SEM of apoptotic cells (**A**., ** P<0.04) and glucose level (**B**., * p< 0.04, ** p< 0.02) and asterisks indicate statistical significance between younger and older cohort.

In response to immune activation by pathogens or by TLR stimulation, the PMN apoptosis program is delayed and the cells may persist at the site of infection for up to 6 hours [26, 29]. The age-related decrease we have noted in TLR1 expression and function may similarly result in a reduced ability to delay apoptosis in PMN of older adults. To assess this, we measured viability and the apoptosis marker Annexin V by flow cytometry in PMN from younger and older age groups after 18 hours in the absence or presence of stimulation (Fig. 5A). Without stimulation, PMN of both groups reached 38-50% positive for markers of apoptosis by 18 hr, with PMN from older donors showing higher levels of apoptotic cells as noted previously [30]. In response to stimulation, although the rate of apoptosis was reduced in PMN from both age groups, PMN from older adults still had higher rates of apoptosis than those of young adults. This age-related difference in rate of apoptosis reached significance when PMN were stimulated through TLR1(Fig. 5A, p< 0.04), reflecting the downstream effects of reduced expression of TLR1, and approached significance when stimulated through TLR4 (Fig. 5A, p=0.06).

Although short-lived, PMN are tremendously active cells, and with few mitochondria, they rely primarily on glycogen stores to support their activity [31]. At an inflammatory site, PMN have been shown to dramatically increase their content of glycogen by up to ten fold, presumably to support the high metabolic requirements of their immune-mediated program [31]. When we measured glucose accumulation in PMN using 2-NBDG, a fluorescent glucose marker, we noted somewhat lower levels of glucose in PMN of older adults at baseline that did not reach statistical significance (Fig 5B, ns). In response to stimulation, as expected, higher levels of glucose were detected in PMN of both age groups and accumulation of fluorescent glucose was higher across all stimulants as compared to untreated PMN (Fig. 5B, p<0.001). Notably, significantly lower levels of glucose accrued in PMN of older adults, particularly when stimulated through the TLR1 pathway (Fig. 5B, p< 0.02), and somewhat less through stimulation the TLR4 pathway (Fig. 5B, p<0.04).

## DISCUSSION

Multiple impairments in innate and adaptive immune responses contribute to reduced immune responses in the elderly [1]. The aim of the present study was to investigate TLRs on PMN from older adults to determine expression levels and functional efficiency of receptors that are key to triggering antimicrobial host defense responses [13, 14]. We found that PMN from older donors express lower levels of TLR1 -- also noted on monocytes and dendritic cells [15, 18]-- which leads to reduced responses to TLR1 stimulation, reduced increases of adhesion molecules (integrins CD11b and CD18), and reduced production of the chemokine IL-8, mediated by reduced levels of activated p38 MAP kinase. Notably, these deficiencies are exacerbated in the frail elderly [32]. Although our study does not distinguish between whether the reduced expression of TLR1 alone accounts for the reduction in PMN function, or whether the remaining TLR1 may be impaired, lower efficiency of this critical pathway in aging is likely to play an important role in multiple PMN functions. In particular, recent reports have shown an association of reduced surface expression of TLR1 with increased susceptibility to *M. tuberculosis* [33].

Studies of the mechanisms of aging suggest that dysregulation of glucose metabolism may underlie many aspects of senescence and shortened lifespan [6, 7]. This is particularly relevant for PMN, which require robust energy homeostasis for the regulation of their function [34], with the decreased glucose response of PMN in aging resembling insulin resistance. Notably, decreased phagocytosis, killing of bacteria, and production of reactive oxygen species have long been observed in PMN from diabetic patients or in animal models of diabetes, which can be reversed by insulin treatment to restore glucose balance [35, 36]. Similarly, dysregulation of glucose has also been noted in PMN from patients with glycogen storage disease type 1b, whose neutrophils are reduced in number and function and show higher rates of apoptosis that were not responsive to G-CSF [37]. We have identified dramatic effects of aging on PMN accumulation of glucose stores, which are essential to fuel anti-microbial activity.

Evaluation of deficiencies in aging may in fact reveal adaptive strategies for successful survival. In our study, the older subjects (average age 74.5 years) remain healthy into old age, suggesting that the TLR1-mediated reduction in PMN function reported here, one of multiple deficiencies noted in immunosenescence [1], may be relevant to surviving other health complications, such as cancer or autoimmune diseases. Recent theories of aging and evidence that caloric restriction enhances longevity suggest paradoxically that reduction in anabolic processes may be beneficial to survival [38]. Caloric restriction is mediated in part by the TOR (target of rapamycin) pathway -- itself under the control of the circadian clock [39]— and while rapamycin inhibition of TOR signaling may be beneficial for longevity, during acute infection it can lead to inappropriate immune responses and increased tissue destruction [40]. Thus successful aging reflects a complex balance between lifespan extension and effective immune responses.

Nevertheless, the deficiency in bioenergetics in PMN described here may underlie several impairments noted in PMN function in aging and may also contribute to enhanced susceptibility to infections, pulmonary congestion, or recovery from burn injury noted in aging [9, 41, 42]. We and others have previously shown that PMN can act as a reservoir for bacterial [43, 44] or viral pathogens [45, 46], and PMN have recently been shown to transport antigen from the dermis to the bone marrow to activate CD8+ T cell responses [47]. It remains unknown whether aging may alter PMN function in elastase function related to colonization of intestinal microbiota or modulation of macrophage TLR4 expression [48, 49], or may contribute to significant PMN-related disease outcomes, including oxidase mediated regulation of chemokine receptors essential for recovery from intestinal inflammation [50]. As the effects of aging on PMN bioenergetics may be amplified in conditions that increase pathogen burdens and may contribute to deficits in the initiation of adaptive responses, our study suggests a potential therapeutic strategy to enhance innate immunity [6].

## EXPERIMENTAL PROCEDURES

### Study Subjects

Heparinized blood from healthy volunteers was obtained with written informed consent under a protocol approved by the Human Investigations Committee of Yale University School of Medicine. Study participants had no acute illness, and took no antibiotics or non-steroidal anti-inflammatory drugs within one month of enrollment. Demographic characteristics of subjects were collected at enrollment. Young adults (n=38) were aged 25.9 years (range 22-30), 55.3% female and 76.3% white. Older adults (n = 40) were aged 74.5 years (range 65-90), 60% female and 92.5% white (Table 1). Subjects were not statistically different for gender in this study. Self-reported information included demographic data, height, weight, medications, and comorbid conditions; immunocompromised individuals were excluded as described previously [17].

### Cell stimulation and flow cytometry

For TLR detection, whole blood (180 μL/tube) was lysed by BD FACS Lysing Solution (BD Biosciences, CA) and PMN were stained with antibodies for lineage markers and TLRs as follows: APC-conjugated CD15, V500-conjugated CD45 (BD Biosciences, CA), PE-conjugated TLR 1, 4 and FITC-conjugated TLR2 (eBioscience, CA) for 30 min at 4°C. The immunostained cells were washed with wash solution and fixed with 1% paraformaldehyde. For stimulation assays, aliquots of whole blood were plated in 96-wells plate (180 μl/well) and incubated for 15 min in medium alone or with TNFα (20 ng/ml; R&D Systems, MN), or ligands for TLR 1 /2 (Pam3CSK4 5 μg/ml; Invivogen, CA), TLR2 (Pam2CSK4 1 μg/ml; Invivogen, CA), and TLR4 (LPS 0.5 μg/ml; Sigma, MO). Red blood cells were lysed and cell suspensions were labeled for 30 min at 4 °C with antibodies for PMN markers as follows: PE-CD11b, FITC-CD18, Pacific Blue-CD62L and V500-conjugated CD45 (BD Biosciences, CA) as described previously [32]. Data were acquired using an LSR II instrument (BD Biosciences, CA) and analyzed using FlowJo software (Tree Star, OR).

### Isolation of human PMN and stimulation for cytokine ELISA, apoptosis, and glucose accumulation measurements

PMN were isolated from heparinized blood via dextran density sedimentation and hypotonic lysis of red blood cells as described [51]. Cells were routinely >95% pure and >99% viable. For apoptosis assays, PMN were stimulated for 18 hr in RPMI-1640 with 10% human serum and TNF-α (20ng/ml), LPS (0.5μg/ml), or Pam3CSK4 (5μg/ml) as indicated before labeling with Annexin V and propidium idodide and detection using LSRII. For quantitation of glucose accumulation, PMN were stimulated as above for 2 hours with 500 μM 2-NBDG (Sigma) added concurrently with stimulation; 2-NBDG levels in CD15+ cells were detected using LSRII. Supernatants from stimulated PMN incubations were stored frozen and cytokines were quantified by batch analysis enzyme-linked immunosorbent assays (ELISA) (OptEIA ELISA kit; BD Biosciences, CA).

### Immunoblot analysis

PMNs (1 × 10^6^/ well) were stimulated for 15 min with Pam3CSK4 (5 μg/ml). Cells were harvested using CelLytic M Cell Lysis buffer (Sigma, MO) containing protease inhibitor cocktail as described previously [17]. Immunoblots were probed with anti-phospho-p38 MAPK,anti-p38 MAPK-andanti-β-actin, developed using Amersham ECL Reagents (GE Healthcare), and scanned using Image J software.

### Statistical analysis

Demographic characteristics were compared with Fisher exact tests for gender, race and ethnicity (Hispanic versus non-Hispanic), while age was sampled to differ, thus ranges are provided. The percentage of TLR positive PMN were compared between younger and older adults with t-test accounting for unequal variances. The Mean Fluorescence Intensity of normalized CD11b, CD18 and CD62L surface expression on PMN were analyzed with mixed effects models adjusting for sex and race, ligand, time, ligand by age group interaction, and ligand by time interaction as described [52]. Similar models were used for apoptosis and fluorescent glucose without the time elements. To address human heterogeneity, we used an unstructured covariance structure for each person to have a unique correlation among TLRs and times points. Statistical tests were 2-tailed, and p<0.05 considered significance. Multivariable analyses used SAS version 9.2 (SAS Institute, Cary, NC) and bivariate analyses used Prism 4.03 biostatistics package (Graphpad, CA).
